# Ice Water

**Published:** 1889-09

**Authors:** 


### Ice Water.

In the opinion of the editor of *The Sanitary Volunteer*, the official organ of the New Hampshire Board of Health, there is a great deal of sentiment and many opinions, regarding the use of ice-water, that vanish when the light of reason and experience is turned upon them. The fact is that ice-water, drank slowly and in moderate quantities, is a healthful and invigorating drink. There is no doubt that ice is a great sanitary agent, and every family ought to be provided with it during the warmer months of the year. It is true that the inordinate use of ice-water, or its use under some special conditions and circumstances, is attended with great danger; so is the improper use of any other drink or food. The assumption that iced water is dangerous, and that iced tea, or iced coffee, or iced lemonade is a harmless substitute, is simply a delusion. As the source of danger feared by some is the degree of cold, we fail to see clearly how flavor modifies the effect of temperature. There are some individuals, undoubtedly, who cannot drink ice-water without injury, and who ought never to use it, but to a great majority of persons it is refreshing and healthful. Its use, temperate and discreet, is in no way to be condemned, which cannot be said of some of its substitutes.—*Science*, June 28, 1889.

A law designed to check the evil of the smoking of cigarettes by boys has been enacted in New York State. The chief of police of New York City, on May 30, issued an order, under that law, stating that it is a misdemeanor for dealers to sell to any person, under sixteen years of age, cigarettes or any other form of tobacco. The selling of alcoholic drink to minors is also prohibited by that law.

